# Isolated Lung Perfusion in the Management of Acute Respiratory Distress Syndrome

**DOI:** 10.3390/ijms21186820

**Published:** 2020-09-17

**Authors:** Nathan Haywood, Matthew R. Byler, Aimee Zhang, Mark E. Roeser, Irving L. Kron, Victor E. Laubach

**Affiliations:** Department of Surgery, University of Virginia School of Medicine, Charlottesville, VA 22902, USA; nsh9x@virginia.edu (N.H.); MRB4M@virginia.edu (M.R.B.); AYZ7KR@virginia.edu (A.Z.); MR8BE@virginia.edu (M.E.R.); ILK@virginia.edu (I.L.K.)

**Keywords:** acute respiratory distress syndrome, acute lung injury, inflammation, isolated lung perfusion, therapeutics

## Abstract

Acute respiratory distress syndrome (ARDS) is associated with high morbidity and mortality, and current management has a dramatic impact on healthcare resource utilization. While our understanding of this disease has improved, the majority of treatment strategies remain supportive in nature and are associated with continued poor outcomes. There is a dramatic need for the development and breakthrough of new methods for the treatment of ARDS. Isolated machine lung perfusion is a promising surgical platform that has been associated with the rehabilitation of injured lungs and the induction of molecular and cellular changes in the lung, including upregulation of anti-inflammatory and regenerative pathways. Initially implemented in an ex vivo fashion to evaluate marginal donor lungs prior to transplantation, recent investigations of isolated lung perfusion have shifted in vivo and are focused on the management of ARDS. This review presents current tenants of ARDS management and isolated lung perfusion, with a focus on how ex vivo lung perfusion (EVLP) has paved the way for current investigations utilizing in vivo lung perfusion (IVLP) in the treatment of severe ARDS.

## 1. Acute Respiratory Distress Syndrome

**Figure 1:**
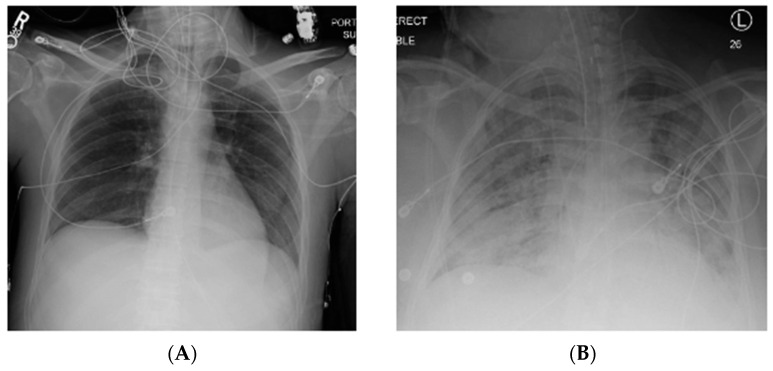
Normal chest X-ray (**A**) compared to that of a patient with acute respiratory distress syndrome (ARDS) (**B**) with bilateral pulmonary infiltrates. R, right; L, left.

Acute respiratory distress syndrome (ARDS) is a severe, life-threatening form of acute lung injury characterized by inflammation, lung permeability, and edema [1,2,3]. Clinical sequelae include significant hypoxia and bilateral infiltrates on chest imaging (Figure 1) [4]. Since the original description by Ashbaugh in 1967, significant advances have been made in the understanding of this disease process [2,5]. However, this syndrome remains common, and is associated with significant morbidity and mortality [1].

Diagnostic criteria of ARDS rely on imaging and other clinical findings. The most recent definition, the Berlin Definition (Table 1), deviates from the previous division of acute lung injury (ALI) and ARDS [6]. Instead, mild, moderate, and severe ARDS severity categories are described based on the PaO_2_/FiO_2_ ratio. Additional tenants include respiratory failure not explained by fluid overload or cardiac failure, characteristic imaging findings (Figure 1), and origin of onset within 7 days of symptoms or known clinical insult [6]. Most common etiologies of ARDS include sepsis, pneumonia, and aspiration, but a number of other less common risk factors exist (Table 2) [1,2]. In addition, new pathogens may emerge that manifest in ARDS, such as the novel coronavirus disease 2019 (COVID-19) [7].

While our understanding of this syndrome has improved over the past half century, it remains a significant burden with a large impact on both the individual and health system. A large recent international evaluation of ARDS in the critical care population demonstrated that 10.4% of intensive care unit (ICU) patients and 23.4% of intubated ICU patients had ARDS [1,8]. In-hospital mortality for the severe subgroup reached as high as 46.1% [1,8]. A recent analysis of the national inpatient sample from 2006–2014 by Eworuke et al. demonstrated increasing incidence of ARDS and a most recent rate of 193.4 cases per 100,000 population [9]. While a downward trend in in-hospital mortality has been reported, it remained quite high over this time period, with rates for sepsis, shock, and pneumonia approximating 40% [9]. In those who physically recover, high risks of cognitive dysfunction, muscular weakness, depression, and post-traumatic stress disorder have been reported [10]. Additionally, the management of patients with ARDS is often resource-intensive (prolonged ventilation and ICU stay) resulting in significant economic impact [11].

Identification and appropriate treatment of the underlying cause of ARDS is essential (e.g., antibiotics, resuscitation, and source control for sepsis) [1]. Beyond this, the current management of ARDS is largely supportive in nature, with basic tenants including lung protective ventilation, conservative fluid management, neuromuscular blockade, and prone positioning for severe disease [1,4]. The use of pharmacologic agents, such as inhaled vasodilators and corticosteroids, can be considered, but their use is controversial and not widely accepted [1,4]. In severe, refractory ARDS, the addition of extracorporeal assistance may provide some benefit [1,4,12].

Lung-protective ventilation with low tidal volumes may cause hypercapnia and associated respiratory acidosis. This is, to some degree, an accepted consequence, leading to a concept known as permissive hypercapnia. Beneficial effects of hypercapnia have been demonstrated, including reduction in pulmonary inflammation and oxidative stress [13,14]. However, recent evidence has emerged suggesting negative impacts of hypercapnia on lung tissue repair and alveolar fluid clearance [13,15]. As such, extracorporeal CO_2_ removal with venovenous extracorporeal membrane oxygenation (ECMO) has been utilized as an alternative means of improving hypercapnia and gas exchange in severe ARDS [12,13]. However, it carries the typical risks associated with extracorporeal circulation [13]. While early evidence has suggested improved outcomes in patients with severe ARDS managed with ECMO [16], a recent international, randomized clinical trial evaluating the use of routine ECMO in severe ARDS showed no difference in mortality compared to conventional ventilatory management with ECMO as a rescue therapy [17]. For this reason, the routine use of ECMO in severe ARDS remains controversial. Recent guidelines suggest consideration of venovenous ECMO in severe ARDS when PaO_2_/FiO_2_ < 80 mmHg despite optimal ARDS treatment, including neuromuscular blockade, high positive end expiratory pressure (PEEP), and prone positioning [18].

The last half decade has produced numerous advances regarding the previously described management paradigm of ARDS, and outcomes have improved over this period. However, mortality remains high, with reports in severe subgroups greater than 40% [1,8]. Given this, and the continued emergence of pathogens causing severe ARDS, such as COVID-19, development and breakthrough of new treatment modalities is crucial [7].

## 2. Isolated Lung Perfusion and ARDS

Because current treatment strategies in ARDS largely rely on supportive measures to stabilize and allow for innate lung recovery over time, there is opportunity for targeted therapeutic approaches to limit disease severity and improve outcomes. Isolated lung perfusion is an active area of investigation that may ultimately serve in this role. First studied in 1987, isolated lung perfusion was initially implemented in an ex vivo fashion for donor lungs prior to transplant [19]. Coined ex vivo lung perfusion (EVLP), it has since undergone numerous advances, leading to its current clinical use in donor lung evaluation and in the reconditioning of marginal donor lungs prior to transplant [20]. The ability of EVLP to rehabilitate lungs injured in a porcine sepsis model [21] has provided the basis for a similar application—the use of isolated lung perfusion in vivo in the management of ARDS. Here, early animal studies have demonstrated the ability of in vivo lung perfusion (IVLP) to rehabilitate sepsis-induced ARDS [22]. Below, we review the history and current evidence for isolated lung perfusion techniques, with a focus on how EVLP has provided the basis for and led to investigations into the use of IVLP for the treatment of ARDS. Relevant works included in our review were identified from PubMed, using the following search terms: ALI, ARDS, isolated lung perfusion, EVLP, and IVLP.

## 3. Ex Vivo Lung Perfusion (EVLP) History

Primary graft dysfunction (PGD) is the result of severe ischemia-reperfusion injury following lung transplant that can result in detrimental early and late outcomes [23]. Surgeons are often conservative in donor lung selection, as lesser quality lungs increase the likelihood of PGD development [24]. It has been reported that only 15–20% of lungs from multiorgan donors are deemed usable for transplantation [24,25,26]. The unfortunate consequence of this is revealed in lung waitlist mortality—reported as high as 17.2 deaths per 100 waitlist years [27]. One strategy to extend donor lung availability is through the use of EVLP.

Clinical application of EVLP was first described by Hardesty et al. in 1987 [19,26]. Since this time, numerous advances have been made, many of which are due to the work of Steen and colleagues, who developed a lung-specific perfusion solution (Steen solution) and first utilized EVLP in donation after cardiac death (DCD) lungs in 2000 [28,29]. Following this achievement, there was a body of work in the early 2000s, with additional contributions by Steen et al. and Wierup et al., demonstrating the ability of EVLP as a clinical platform for continued assessment and rehabilitation in lungs considered unusable at the time of initial evaluation [30,31]. The Toronto Lung Transplant Group then demonstrated the efficacy of extended-duration EVLP in a landmark study published in the *New England Journal of Medicine* in 2011 [20]. Here, they described 20 cases in which lungs were initially “not considered suitable for transplantation” that underwent reevaluation during a 4 h period of EVLP and were ultimately successfully transplanted. The incidence of PGD within 72 h was 15% in the EVLP group compared to 30% in the control group (*p* = 0.11), and there were no significant differences for any secondary endpoints, including ECMO, post-transplantation ICU days, 30 day mortality, *p*/F ratio, or any severe adverse events directly attributable to EVLP [20]. In the last decade, the clinical use of EVLP has become more widespread, and is now used in many large lung transplant centers in North America, Europe, and Australia [32]. A recent multicenter prospective clinical trial (NOVEL) examining the use of EVLP has demonstrated similar early outcomes and one-year survival compared to patients that underwent transplantation with standard criteria donor lungs [33,34].

## 4. EVLP Technique

**Figure 2:**
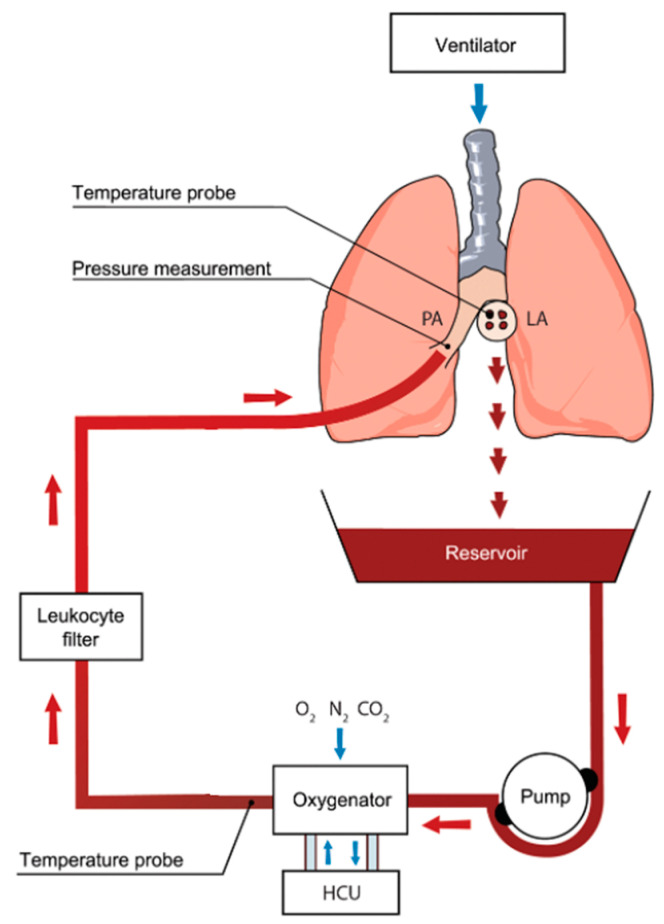
Ex vivo lung perfusion (EVLP) circuit showing core components. Oxygenated blood is drained from the left atrium (LA). It is pumped through an oxygenator where gas exchange occurs. Deoxygenated blood is then subjected to a leukocyte-reducing filter and returned to the main pulmonary artery (PA). HCU: heating cooling unit. Used with permission [37]. Red arrows indicate direction of perfusate flow; blue arrows indicate direction of gas delivered by ventilator, sweep gas, and flow around the heating cooling unit.

EVLP utilizes physiologic, normothermic perfusion of donor lungs [35]. This allows for a period of lung assessment during which direct examination, imaging, bronchoscopy, and blood gas analysis can be performed [26]. A general circuit for EVLP is shown in Figure 2. The device is made up of two main components. The first is a ventilator to provide oxygen to the donor lungs, and the second is a circuit that drives perfusion, deoxygenates, and filters the perfusate [36]. The perfusate is deoxygenated using a gas exchange membrane with a sweep gas consisting of nitrogen, CO_2_, and O_2_ [23], which is then filtered using a leukocyte filter prior to advancing into the pulmonary artery [35,36]. The perfusate is drained from the left atrium into a reservoir prior to repeating the sequence [36].

While there are a number of ex vivo perfusion systems under investigation, the two most common systems used in clinical practice are the XVIVO Perfusion System (XPS) (XPS Perfusion, Goteborg, Sweden) and the Organ Care System (OCS) (Transmedics, Andover, MA, USA) [23]. A comparison of these two systems is shown in Table 3. While both systems utilize the normothermic perfusion of donor lungs, the specific perfusate utilized is different. XPS utilizes an acellular perfusate (STEEN Solution), which contains albumin, dextran, and electrolytes, and often includes additives such as steroids and antibiotics [21,23]. The perfusate used for OCS includes a cellular component (packed red blood cells) as well as OCS solution, which is composed of a low-potassium dextran solution and additives, such as steroids, glucose, bicarbonate, and antibiotics [23]. Another distinction between these systems is that OCS is portable while XPS is static [23,38]. The OCS system integrates all components into a compact unit small enough to fit in a passenger seat in a car or plane [23]. Given the portability, lungs can be instrumented onto the OCS system at the donor hospital following cold antegrade and retrograde flush. This has the theoretic benefit of minimizing cold ischemic time during transport to the recipient hospital [23,26]. Alternatively, XPS is typically maintained at the recipient hospital, and lungs are instrumented onto the system upon arrival—following a period of cryopreservation [23]. Arterial blood gasses are performed hourly using XPS and are monitored continuously with OCS. If deemed acceptable, lungs are flushed with cold perfusate and kept cool prior to the implantation procedure [23].

## 5. Investigational Therapies During EVLP

EVLP has undergone a transformation from an assessment and diagnostic tool to a therapeutic platform that also allows for active lung rehabilitation. This platform provides an ideal environment to deliver targeted drug therapy for lung rehabilitation, as it allows the opportunity to re-evaluate function to confirm positive treatment effect, and allows for targeted treatment of the lung, minimizing the risk of treatment side effects that may preclude systemic administration of therapeutic agents. One target of pharmacological agents in EVLP has been minimizing inflammation and the reduction of pulmonary edema. Numerous investigational agents have shown promise. For example, the administration of aerosolized exogenous catecholamines into the distal airspaces during EVLP has been demonstrated to enhance the clearance of pulmonary edema, resulting in better graft oxygenation, pulmonary compliance, and reduced pulmonary vascular resistance [39,40,41]. This effect is not isolated to aerosolized delivery, as perfusion with a short-acting selective beta-2 adrenergic receptor agonist has also been associated with lower pulmonary artery pressures and better lung mechanics [42].

Our laboratory and others have recently studied EVLP as a platform to recondition lungs via pharmacologic treatment during ex vivo perfusion. Using both murine and porcine models, we have demonstrated that the addition of a selective adenosine 2A receptor (A2AR) agonist to the EVLP perfusate is associated with less pulmonary edema, lower levels of pro-inflammatory cytokines, and improved lung function [43,44]. Similarly, utilizing a porcine DCD model, our lab has demonstrated that delivery of A2AR agonist during EVLP increased the likelihood of successful transplantation following prolonged periods of cold preservation [45]. The addition of a selective adenosine 2B receptor antagonist to the EVLP perfusate has also been associated with improved lung function in both murine and porcine models [46,47]. Numerous other pharmacologic agents administered using the EVLP platform have shown promise in mitigating the pulmonary inflammatory response, including, but not limited to, sphingosine-1-phosphate [48], neutrophil elastase inhibitor [49], and alpha-1-antitrypsin [50]. Alpha-1-antitrypsin treatment was found to significantly reduce pulmonary edema, pulmonary cell apoptosis, and pro-inflammatory cytokine levels (IL-1α and IL-8) in the perfusate [50].

Similar to drug therapy, several studies have demonstrated that gene therapy coupled with EVLP can repair injured lungs before transplantation. Cypel et al. showed that delivery of an adenoviral vector encoding human IL-10 (AdhIL-10), an anti-inflammatory cytokine, to human lungs improved arterial oxygen pressure and vascular resistance during EVLP, concluding that delivery of AdhIL-10 can improve lung function [51]. Yeung et al. later showed that ex vivo delivery of AdhIL-10 to lungs is superior to in vivo delivery, in that it leads to less vector-associated inflammation and provides superior post-transplant lung function [52].

## 6. Molecular and Cellular Changes During EVLP

A variety of recent studies have begun to evaluate molecular and cellular changes that occur during EVLP. Using a porcine model, Tavasoli et al. showed that EVLP resulted in reduced concentrations of nitric oxide metabolites and L-citrulline in lung tissue [53]. In addition, the ratio of L-ornithine over L-citrulline, a marker of the balance between L-arginine metabolizing enzymes, was increased in the EVLP group, and expression of both arginase isoforms was increased during EVLP. These data suggest that EVLP induces a shift of the L-arginine balance towards arginase, leading to nitric oxide deficiency in the lung. Using a rat model of EVLP, Lonati and colleagues described a remarkable anti-inflammatory response during EVLP, including the activation of protective and anti-apoptotic pathways [54]. They also detected resolution factors in perfused, uninjured lungs, including transcripts that encode for feedback inhibitors of Toll-like receptors and cytokine signaling, such as inhibitors of nuclear factor-κB (NF-κB) signaling IκB (inhibitor of κB), IL-1 receptor antagonist 1, lL-1 decoy receptor, and nonfunctional interleukin 1 receptor-associated kinase-M. Importantly, their data obtained in uninjured lungs was confirmed in perfused injured (DCD) lungs. These results led Lonati and colleagues to conclude that the EVLP molecular signature is very similar to the pattern induced by ischemic preconditioning [54].

To identify potential biomarkers during EVLP, Hsin et al. used a metabolomics approach in a clinical study to identify a small panel of metabolites in EVLP perfusate that were highly correlated with the development of PGD after transplant [55]. In another clinical EVLP biomarker study, Hashimoto et al. demonstrated that levels of M30 (indicative of epithelial apoptosis) and high mobility group box 1 (HMGB-1, related to cell death and inflammation) protein in the EVLP perfusate correlated with PGD after lung transplantation, and might therefore be useful biomarkers to improve donor lung assessment during EVLP [56]. A recent study by Elgharably et al. showed that two microRNAs (miR-17 and miR-548b) were significantly upregulated in the alveolar epithelial cells of human lungs that underwent cold ischemia and EVLP [57]. Both miR-17 and miR-548b have expected target genes related to lung injury and share a number of mutual targets, suggesting that miR-17 and miR-548b may interact at some level in the signaling pathway and potentially provide novel therapeutic targets [57]. Yeung and colleagues examined gene expression changes in human lungs during 12 h of EVLP, and found that, despite increases in endothelial markers of inflammation, circulating, leukocyte, cell-specific gene expression fell during EVLP [58]. These results suggest that perhaps the mechanisms underlying the benefit of EVLP are nonspecific and related to innate recovery capabilities of the lung. Finally, a recent study by Wong et al. performed a retrospective transcriptomics analysis of DCD lungs with or without EVLP, and showed that pathways associated with leukocyte function, such as phosphatidylinositol biosynthesis, phospholipase C signaling, cholesterol biosynthesis, protein targeting to vacuoles, and Golgi vesicle trafficking were all downregulated in lungs after EVLP [59]. These results support those of Yeung et al. above [58], which inferred that passenger leukocytes are depleted during EVLP.

The ability of EVLP to rehabilitate injured, marginal lungs prior to transplantation has led to investigation into other forms of lung injury—namely, ARDS. Our laboratory has demonstrated the ability of EVLP to rehabilitate ARDS in a porcine sepsis model [21]. In this study, intravenous lipopolysaccharide (LPS) was used to generate a systemic inflammatory response with associated ARDS [21]. Lungs that were subjected to EVLP with Steen solution demonstrated improved oxygenation and compliance compared to the control (no EVLP) [21]. This finding provided the basis for investigation of a similar in vivo technique, IVLP, in the management and rehabilitation of ARDS.

## 7. In Vivo Lung Perfusion (IVLP) History

In vivo lung perfusion (IVLP) was first investigated in the 1980s as a method for delivering high-dose chemotherapy [60,61]. IVLP involves isolation and placement of cannulas into the pulmonary artery and veins of a single lung in vivo, so that its perfusion is removed from systemic circulation. This allows for the delivery of high dose medication to the lung parenchyma while limiting adverse systemic effects. In this way, much higher doses of chemotherapy could be used to treat lung cancer than would have otherwise been tolerated systemically. Multiple clinical trials investigating the utility of IVLP in the treatment of lung cancer have shown increased survival benefit [62,63]. These studies, coupled with studies showing that EVLP can rehabilitate sepsis-induced lung injury [21], have provided the basis and rational for investigating the use of IVLP to rehabilitate end-stage lung injury from ARDS. Currently, investigations into the use of IVLP for the rehabilitation of ARDS have been limited to swine animal models, as described below.

## 8. IVLP Technique

Unlike EVLP, which perfuses previously resected donor lungs, IVLP provides isolated lung perfusion to lungs that remain inside of a host. IVLP investigations have achieved this via a sternotomy or thoracotomy approach [22,64]. Prior to beginning the procedure, an injurious model is used to establish lung injury, which can be achieved by various protocols, including intravenous LPS, surfactant washout model, intravenous oleic acid, or gastric aspiration [65,66]. Our laboratory has established a systemic lung injury model in swine using an LPS infusion administered at 50 µg/kg over 2 h to establish ARDS, defined as a *p*/F ratio less than 300 mmHg. This model creates a reproducible injury and simulates the increased capillary permeability observed in a septic response. Due to the systemic, hemodynamic instability associated with this injury model, we performed IVLP via a sternotomy, in order to provide necessary cardiopulmonary support via central, venoarterial ECMO [22].

**Figure 3:**
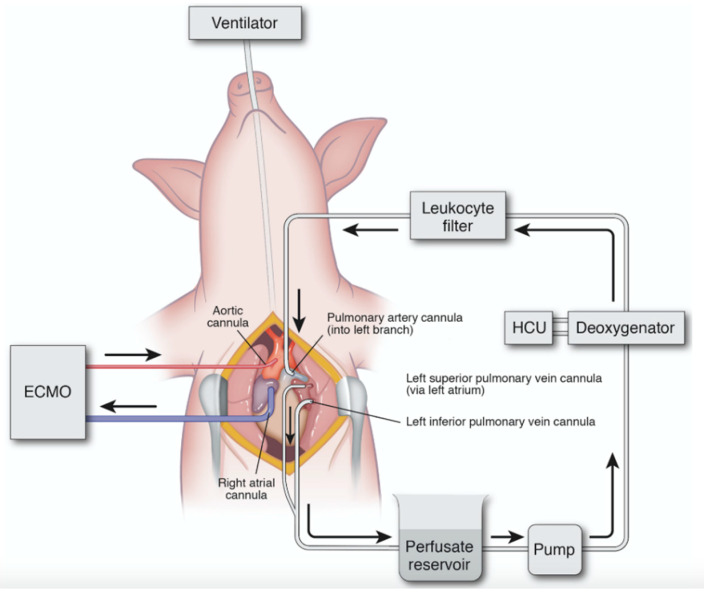
Diagram of porcine in vivo lung perfusion (IVLP) circuit. Inflow is via direct cannulation of the left pulmonary artery, and outflow is direct cannulation of the superior and inferior pulmonary vein. Flow is maintained at 8% estimated cardiac output, and the circuit is primed with Steen solution. Circuit includes reservoir, pump, deoxygenator, and leukocyte filter. ECMO: extracorporeal membrane oxygenation; HCU: heating–cooling unit. Used with permission [22]. Arrows indicate direction of perfusate flow in IVLP circuit and venous outflow/arterial inflow in ECMO circuit.

Our IVLP investigations utilized perfusion of the left porcine lung, due to its optimal venous anatomy for an open approach. The left pulmonary artery and superior and inferior veins were circumferentially dissected, and cannulas were placed into the vessels, as outlined in Figure 3. These cannulas are circumferentially secured so as to isolate the left lung from systemic circulation. The cannulas are connected to an IVLP circuit that is designed similarly to that used for cardiopulmonary bypass. The circuit uses a special gas mixture and a membrane deoxygenator, which provides physiologic levels of carbon dioxide and removes oxygen from the Steen perfusate. After the perfusate circulates through the lung, the lung’s oxygenation and ventilation capacity are evaluated by blood gas analysis at predetermined intervals. The back pressure on the pulmonary venous drainage was maintained at between 0 to +5 mmHg by adjusting the height of the hard-shelled cardiotomy reservoir. After completion of a predetermined IVLP perfusion period (2 or 4 h), the cannulas were removed, and the lung was allowed to reperfuse back into systemic circulation.

## 9. Investigations of IVLP in ARDS

Multiple studies have investigated the use of IVLP for the delivery of isolated, high-dose chemotherapy to the lung. The duration of this treatment has been for 30 min [62]. However, the use of IVLP to treat ARDS has utilized longer treatment (perfusion) times. In 2014, dos Santos and colleagues evaluated the use of prolonged IVLP in a large animal study, where they delivered IVLP for 4 h via a thoracotomy to six swine, followed by a 4 h reperfusion period [64]. Here, they demonstrated that using IVLP for this duration is feasible and safe, and there was no change in lung function parameters (oxygenation and compliance) or histologic evidence of acute lung injury [64].

In 2018, our laboratory was the first to investigate the use of IVLP to rehabilitate sepsis-induced ARDS [22]. After undergoing an LPS infusion and confirmation of ARDS, 4 h of left-lung IVLP with Steen solution was performed in eight swine. The right lung served as an internal control, and was compared the IVLP-treated left lung. After the IVLP treatment period, the animal was decannulated from IVLP and allowed to reperfuse for 4 h. Over the course of the experiment, the treated left lungs demonstrated improved oxygenation performance from baseline when compared to the right lung controls. Additionally, total lung compliance was increased. The mechanism behind these improvements may be due in part to the observed decrease in levels of tumor necrosis factor alpha (TNF-α) and interferon gamma (IFN-γ) in the treated left lungs. Additionally, there was evidence of decreased pulmonary edema, demonstrated by lower wet-to-dry weight ratios in the treated left lungs. Finally, there was decreased expression of the cellular adhesion molecules vascular cell adhesion molecule 1 (VCAM-1) and intercellular adhesion molecule 1 (ICAM-1). These data suggest that there was decreased transmigration of leukocytes, which resulted in decreased histologic evidence of inflammation in the IVLP-treated lungs [22]. 

Most recently, we performed a study comparing the previously used IVLP perfusion time of 4 h to 2 h of IVLP perfusion [67]. Similar to our previous study, eight adult swine underwent LPS infusion to induce ARDS, and then were randomized to either 4 h IVLP (*n* = 4) or 2 h IVLP (*n* = 4) treatment groups. The results demonstrated that 2 h of IVLP outperformed 4 h of IVLP when evaluating each lung’s oxygenation capacity and total lung compliance. Similar to the study by Mehaffey et al. [22], this was likely due to a reduction in pulmonary edema, as indicated by improved wet-to-dry weight ratios. We also observed decreased expression of the pleiotropic cytokine IL-6 [67]. This cytokine is characteristically elevated in the hyperinflammatory subphenotype of ARDS, and has been targeted by monoclonal antibodies in the treatment of ARDS associated with COVID-19 [68,69]. Together, these physiologic and biochemical improvements in the treated lungs allowed more animals to successfully wean from venoarterial ECMO support in the 2 h group (three of four) than in the 4 h group (two of four).

These two studies shared some limitations, such as they were performed in farm-raised animals that may have physiologic variability and were comprised of low group sizes. Because we have not yet determined if the results with the LPS model of ARDS are translatable to other etiologies of ARDS (e.g., pulmonary contusions, massive transfusion reaction, aspiration, etc.), the results may not be generalizable.

## 10. Clinical Translation and Future Direction of IVLP

**Figure 4:**
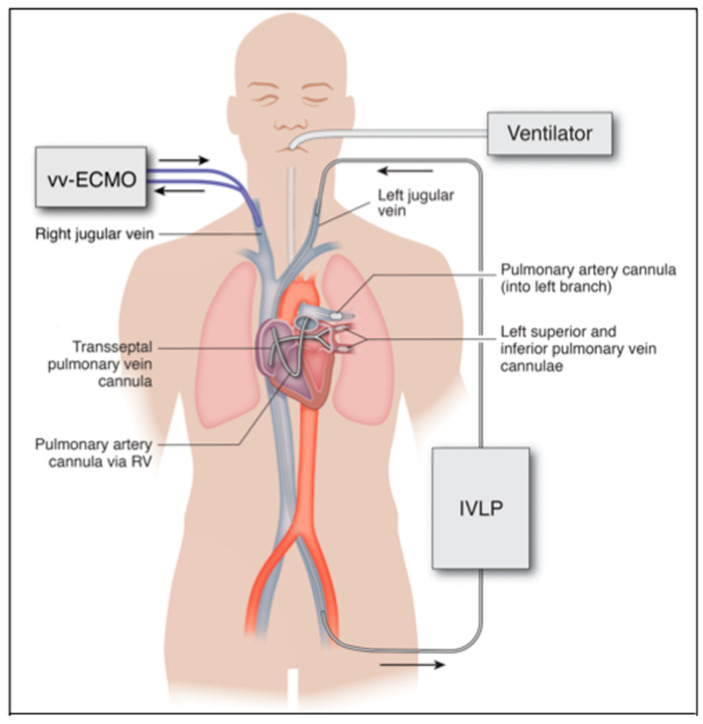
Proposed percutaneous IVLP approach in humans. Inflow through left pulmonary artery cannula inserted from the left internal jugular vein and outflow from left pulmonary veins via bifurcated cannula placed transseptal from the left femoral vein. Arrows indicate direction of flow in ECMO and IVLP circuits.

The invasive sternotomy and thoracotomy approaches used in IVLP investigations are currently much too invasive to use in a critically ill, unstable patient. The future of IVLP in the treatment of ARDS will be contingent upon the advancement of catheter-based technologies that can be translated into a percutaneous platform. This will provide a minimally invasive application of IVLP in lung rehabilitation via catheters placed into peripheral vessels and threaded over a wire into the pulmonary artery and veins, instead of large surgical incisions (Figure 4).

There remains a myriad of questions to be answered, in order to establish the optimal protocol for IVLP in the treatment of ARDS. We have demonstrated improved pulmonary function following IVLP and a 4 h reperfusion period [22,67]. However, the optimal timing of IVLP, as well as the long-term effect of IVLP on lung function beyond 4 h of reperfusion, is yet to be determined. The early exudative phase of ARDS is characterized by innate inflammatory cell activation, resulting in damage to the alveolar epithelium and capillary endothelium [1,2,4]. While further studies are needed to determine the optimal timing of IVLP, it is likely within this early phase of ARDS that IVLP will provide the most benefit. Less damage in this phase would decrease the negative impact of innate recovery pathways that can result in lung fibrosis. Limiting the progression to fibrosis is critical for recovery, as this final fibrotic phase is associated with prolonged ventilation and increased mortality [1,4]. Additionally, the rehabilitative effect of IVLP on pulmonary function in different ARDS models remains to be evaluated. These are important questions that warrant further investigation prior to translation into human applications.

Causes of ARDS can be grouped into two broad categories: those that cause direct lung injury and those that cause an indirect lung injury. The latter often results from the deleterious impact of a systemic inflammatory response, such as sepsis. The pathophysiology of these two categories differs, and while we have demonstrated the beneficial impact of IVLP in a sepsis model, we have yet to determine the impact in a direct lung injury model, such as aspiration or ventilator-induced lung injury. The benefit of IVLP in a sepsis model is multifactorial, but a large part is due to the protective effect of isolated lung perfusion in the setting of systemic inflammation. Future use of IVLP will extend beyond simply the perfusion of Steen solution to ameliorate lung injury and decrease pulmonary edema. Similar to EVLP, the technique of IVLP can be advanced and used as a platform for the delivery of a myriad of lung-specific drug therapies. This avenue may provide additional benefit in more direct causes of lung injury, such as gastric aspiration, pneumonia, and ventilator-induced lung injury. With administration to lung circulation that is isolated from systemic circulation, the delivery of higher-dose drug therapies than would otherwise be tolerated from systemic side effects would be possible. These therapies may include powerful antimicrobials, antivirals, immunomodulators, stem cells, genetic therapies, and any combination thereof to target the exact etiology of a patient’s ARDS and limit the local injurious inflammatory response [70]. ARDS remains associated with high rates of morbidity and mortality, and advances in management will be critical to improve outcomes and decrease the drastic impact on healthcare resource utilization [1,8,11]. In vivo isolated lung perfusion is a promising investigational method for lung rehabilitation in severe ARDS.

## Figures and Tables

**Table 1:** Diagnosis of acute respiratory distress syndrome: Berlin criteria [6].

Timing	Occurs Within 7 Days of Known Insult or the Initial Decline in Respiratory Status
Imaging	Bilateral opacities noted on either CXR or CT scan that are not otherwise explained by fluid overload or cardiac failure
Categorization	Mild: PaO_2_/FiO_2_ = 200–300 mmHg Moderate: PaO_2_/FiO_2_ = 100–200 mmHg Severe: PaO_2_/FiO_2_ < 100 mmHg Assessed with PEEP 5 or greater

CXR: chest X-ray, CT: computerized tomography, PaO_2_: partial pressure of arterial oxygen, FiO_2_: fraction of inspired oxygen, PEEP: positive end expiratory pressure.

**Table 2:** Acute respiratory distress syndrome risk factors.

Sepsis
Pneumonia
Aspiration
Ventilator-induced lung injury
Pancreatitis
Trauma and burn Injury
Blood product administration
Cardiopulmonary bypass
Ischemia-reperfusion injury following lung transplantation
COVID-19 infection

**Table 3:** Comparison of the two most commonly used EVLP systems.

Parameter	OCS	XPS
Perfusate	RBC + OCS solution	Steen solution
Perfusion goal	1.5–2.0 L/min	40% cardiac output
Portable?	Yes	No
Starting temperature	34 °C	32 °C
Target temperature	37 °C	37 °C
Respiratory rate	12	7
Tidal volume	6 mL/kg	7 mL/kg
PEEP	5 cm H_2_O	5 cm H_2_O
FiO_2_	21%	21%

OCS: Organ Care System, XPS: XVIVO Perfusion System, RBC: red blood cell, PEEP: positive end expiratory pressure, FiO_2_: fraction of inspired oxygen.

## References

[1] Thompson B.T., Chambers R.C., Liu K.D. (2017). Acute Respiratory Distress Syndrome. N. Engl. J. Med..

[2] Matthay M.A. (1996). The Acute Respiratory Distress Syndrome. N. Engl. J. Med..

[3] Mokra D., Mikolka P., Košútová P., Mokry J. (2019). Corticosteroids in Acute Lung Injury: The Dilemma Continues. Int. J. Mol. Sci..

[4] Umbrello M., Formenti P., Bolgiaghi L., Chiumello D. (2016). Current Concepts of ARDS: A Narrative Review. Int. J. Mol. Sci..

[5] Ashbaugh D., Bigelow D.B., Petty T., Levine B. (1967). Acute respiratory distress in adults. Lancet.

[6] Ranieri V.M., Rubenfeld G., Thompson B.T., Ferguson N.D., Caldwell E., Fan E., Camporota L., Slutsky A.S. (2012). Acute Respiratory Distress Syndrome. JAMA.

[7] Guan W.-J., Ni Z.-Y., Hu Y., Liang W.-H., Ou C.-Q., He J.-X., Liu L., Shan H., Lei C.-L., Hui D.S. (2020). Clinical Characteristics of Coronavirus Disease 2019 in China. N. Engl. J. Med..

[8] Bellani G., Laffey J., Pham T., Fan E., Brochard L., Esteban A., Gattinoni L., Van Haren F.M.P., Larsson A., McAuley D.F. (2016). Epidemiology, Patterns of Care, and Mortality for Patients With Acute Respiratory Distress Syndrome in Intensive Care Units in 50 Countries. JAMA.

[9] Eworuke E., Major J.M., McClain L.I.G. (2018). National incidence rates for Acute Respiratory Distress Syndrome (ARDS) and ARDS cause-specific factors in the United States (2006–2014). J. Crit. Care.

[10] Herridge M., Moss M., Hough C.L., Hopkins R.O., Rice T.W., Bienvenu O.J., Azoulay E. (2016). Recovery and outcomes after the acute respiratory distress syndrome (ARDS) in patients and their family caregivers. Intensiv. Care Med..

[11] Bice T., Carson S.S. (2019). Acute Respiratory Distress Syndrome: Cost (Early and Long-Term). Semin. Respir. Crit. Care Med..

[12] Brodie D., Bacchetta M. (2011). Extracorporeal Membrane Oxygenation for ARDS in Adults. N. Engl. J. Med..

[13] Morales-Quinteros L., Camprubí-Rimblas M., Bringué J., Bos L.T., Schultz M.J., Artigas A. (2019). The role of hypercapnia in acute respiratory failure. Intensiv. Care Med. Exp..

[14] Broccard A.F., Hotchkiss J.R., Vannay C., Markert M., Sauty A., Feihl F., Schaller M.-D. (2001). Protective Effects of Hypercapnic Acidosis on Ventilator-induced Lung Injury. Am. J. Respir. Crit. Care Med..

[15] O’Toole D., Hassett P., Contreras M., Higgins B.D., McKeown S.T.W., McAuley D.F., O’Brien T., Laffey J.G. (2009). Hypercapnic acidosis attenuates pulmonary epithelial wound repair by an NF-kappaB dependent mechanism. Thorax.

[16] Peek G.J., Mugford M., Tiruvoipati R., Wilson A., Allen E., Thalanany M.M., Hibbert C.L., Truesdale A., Clemens F., Cooper N. (2009). Efficacy and economic assessment of conventional ventilatory support versus extracorporeal membrane oxygenation for severe adult respiratory failure (CESAR): A multicentre randomised controlled trial. Lancet.

[17] Combes A., Hajage D., Capellier G., Demoule A., Lavoué S., Guervilly C., Da Silva D., Zafrani L., Tirot P., Veber B. (2018). Extracorporeal Membrane Oxygenation for Severe Acute Respiratory Distress Syndrome. N. Engl. J. Med..

[18] Papazian L., Aubron C., Brochard L., Chiche J.-D., Combes A., Dreyfuss D., Forel J.-M., Guérin C., Jaber S., Dessap A.M. (2019). Formal guidelines: Management of acute respiratory distress syndrome. Ann. Intensiv. Care.

[19] Hardesty R.L., Griffith B.P. (1987). Autoperfusion of the heart and lungs for preservation during distant procurement. J. Thorac. Cardiovasc. Surg..

[20] Cypel M., Yeung J.C., Liu M., Anraku M., Chen-Yoshikawa T.F., Karolak W., Sato M., Laratta J., Azad S., Madonik M. (2011). Normothermic Ex Vivo Lung Perfusion in Clinical Lung Transplantation. N. Engl. J. Med..

[21] Mehaffey J.H., Charles E.J., Sharma A.K., Salmon M., Money D., Schubert S., Stoler M.H., Tribble C.G., Laubach V.E., Roeser M.E. (2017). Ex Vivo Lung Perfusion Rehabilitates Sepsis-Induced Lung Injury. Ann. Thorac. Surg..

[22] Mehaffey J.H., Charles E.J., Schubert S., Salmon M., Sharma A.K., Money D., Stoler M.H., Laubach V.E., Tribble C.G., Roeser M.E. (2018). In vivo lung perfusion rehabilitates sepsis-induced lung injury. J. Thorac. Cardiovasc. Surg..

[23] Loor G. (2019). EVLP: Ready for Prime Time?. Semin. Thorac. Cardiovasc. Surg..

[24] Cypel M. (2012). Isolated lung perfusion. Front. Biosci..

[25] Punch J.D., Hayes D.H., Laporte F.B., McBride V., Seely M.S. (2007). Organ Donation and Utilization in the United States, 1996–2005. Am. J. Transplant..

[26] Pan X., Yang J., Fu S., Zhao H. (2018). Application of ex vivo lung perfusion (EVLP) in lung transplantation. J. Thorac. Dis..

[27] Valapour M., Skeans M.A., Smith J.M., Edwards L.B., Cherikh W.S., Uccellini K., Israni A.K., Snyder J.J., Kasiske B.L. (2017). OPTN/SRTR 2015 Annual Data Report: Lung. Am. J. Transplant..

[28] Steen S., Sjöberg T., Pierre L., Liao Q., Eriksson L., Algotsson L. (2001). Transplantation of lungs from a non-heart-beating donor. Lancet.

[29] Steen S., Liao Q., Wierup P.N., Bolys R., Pierre L., Sjöberg T. (2003). Transplantation of lungs from non-heart-beating donors after functional assessment ex vivo. Ann. Thorac. Surg..

[30] Steen S., Ingemansson R., Eriksson L., Pierre L., Algotsson L., Wierup P., Liao Q., Eyjolfsson A., Gustafsson R., Sjöberg T. (2007). First Human Transplantation of a Nonacceptable Donor Lung After Reconditioning Ex Vivo. Ann. Thorac. Surg..

[31] Wierup P., Haraldsson Å., Nilsson F., Pierre L., Scherstén H., Silverborn M., Sjöberg T., Westfeldt U., Steen S. (2006). Ex Vivo Evaluation of Nonacceptable Donor Lungs. Ann. Thorac. Surg..

[32] Ali A., Cypel M. (2019). Ex-vivo lung perfusion and ventilation. Curr. Opin. Organ. Transplant..

[33] Sanchez P.G., Davis R.D., D’ovidio F., Weyan M.J., Camp P.C., Cantu III E., Griffith B.P. (2013). Normothermic Ex Vivo Lung Perfusion as an Assessment of Marginal Donor Lungs–The NOVEL Lung Trial. J. Hear. Lung Transplant..

[34] Sanchez P., Davis R., D’Ovidio F., Cantu E., Weyant M., Camp P., Griffith B. (2014). The NOVEL Lung Trial One-Year Outcomes. J. Hear. Lung Transplant..

[35] Cypel M., Keshavjee S. (2016). Extracorporeal lung perfusion (ex-vivo lung perfusion). Curr. Opin. Organ. Transplant..

[36] Tane S., Noda K., Shigemura N. (2017). Ex Vivo Lung Perfusion. Chest.

[37] Wallinder A., Ricksten S.-E., Silverborn M., Hansson C., Riise G.C., Liden H., Jeppsson A., Dellgren G. (2013). Early results in transplantation of initially rejected donor lungs after ex vivo lung perfusion: A case-control study. Eur. J. Cardio-Thoracic Surg..

[38] Van Raemdonck D.E., Rega F., Rex S., Neyrinck A. (2018). Machine perfusion of thoracic organs. J. Thorac. Dis..

[39] Kondo T., Chen-Yoshikawa T.F., Ohsumi A., Hijiya K., Motoyama H., Sowa T., Ohata K., Takahashi M., Yamada T., Sato M. (2015). β2-Adrenoreceptor Agonist Inhalation During Ex Vivo Lung Perfusion Attenuates Lung Injury. Ann. Thorac. Surg..

[40] Hijiya K., Chen-Yoshikawa T.F., Kondo T., Motoyama H., Ohsumi A., Nakajima D., Sakamoto J., Ohata K., Takahashi M., Tanaka S. (2017). Bronchodilator Inhalation During Ex Vivo Lung Perfusion Improves Posttransplant Graft Function After Warm Ischemia. Ann. Thorac. Surg..

[41] Sakuma T., Gu X., Wang Z., Maeda S., Sugita M., Sagawa M., Osanai K., Toga H., Ware L.B., Folkesson G. (2006). Stimulation of alveolar epithelial fluid clearance in human lungs by exogenous epinephrine. Crit. Care Med..

[42] Valenza F., Rosso L., Coppola S., Froio S., Colombo J., Dossi R., Fumagalli J., Salice V., Pizzocri M., Conte G. (2012). β-Adrenergic agonist infusion during extracorporeal lung perfusion: Effects on glucose concentration in the perfusion fluid and on lung function. J. Hear. Lung Transplant..

[43] Emaminia A., Lapar D.J., Zhao Y., Steidle J.F., Harris D.A., Laubach V.E., Linden J., Kron I.L., Lau C.L. (2011). Adenosine A_2_A agonist improves lung function during ex vivo lung perfusion. Ann Thorac. Surg..

[44] Stone M.L., Sharma A.K., Mas V.R., Gehrau R.C., Mulloy D.P., Zhao Y., Lau C.L., Kron I.L., Huerter M.E., Laubach V.E. (2015). Ex Vivo Perfusion With Adenosine A2A Receptor Agonist Enhances Rehabilitation of Murine Donor Lungs After Circulatory Death. Transplant..

[45] Wagner C.E., Pope N.H., Charles E.J., Huerter M.E., Sharma A.K., Salmon M.D., Carter B.T., Stoler M.H., Lau C.L., Laubach V.E. (2015). Ex vivo lung perfusion with adenosine A2A receptor agonist allows prolonged cold preservation of lungs donated after cardiac death. J. Thorac. Cardiovasc. Surg..

[46] Charles E.J., Mehaffey J.H., Sharma A.K., Zhao Y., Stoler M.H., Isbell J.M., Lau C.L., Tribble C.G., Laubach V.E., Kron I.L. (2017). Lungs donated after circulatory death and prolonged warm ischemia are transplanted successfully after enhanced ex vivo lung perfusion using adenosine A2B receptor antagonism. J. Thorac. Cardiovasc. Surg..

[47] Huerter M.E., Sharma A.K., Zhao Y., Charles E.J., Kron I.L., Laubach V.E. (2016). Attenuation of Pulmonary Ischemia-Reperfusion Injury by Adenosine A2B Receptor Antagonism. Ann. Thorac. Surg..

[48] Mehaffey J.H., Charles E.J., Narahari A.K., Schubert S., Laubach V.E., Teman N.R., Lynch K.R., Kron I.L., Sharma A.K. (2018). Increasing circulating sphingosine-1-phosphate attenuates lung injury during ex vivo lung perfusion. J. Thorac. Cardiovasc. Surg..

[49] Harada M., Oto T., Otani S., Miyoshi K., Okada M., Iga N., Nishikawa H., Sugimoto S., Yamane M., Miyoshi S. (2015). A neutrophil elastase inhibitor improves lung function during ex vivo lung perfusion. Gen. Thorac. Cardiovasc. Surg..

[50] Lin H., Chen M., Tian F., Tikkanen J., Ding L., Cheung H.Y.A., Nakajima D., Wang Z., Mariscal A., Hwang D. (2018). α 1 -Anti-trypsin improves function of porcine donor lungs during ex-vivo lung perfusion. J. Hear. Lung Transplant..

[51] Cypel M., Liu M., Rubacha M., Yeung J.C., Hirayama S., Anraku M., Sato M., Medin J., Davidson B.L., De Perrot M. (2009). Functional Repair of Human Donor Lungs by IL-10 Gene Therapy. Sci. Transl. Med..

[52] Yeung J.C., Wagnetz D., Cypel M., Rubacha M., Koike T., Chun Y.-M., Hu J., Waddell T.K., Hwang D.M., Liu M. (2012). Ex Vivo Adenoviral Vector Gene Delivery Results in Decreased Vector-associated Inflammation Pre- and Post–lung Transplantation in the Pig. Mol. Ther..

[53] Tavasoli F., Liu M., Machuca T.N., Bonato R., Grant D.R., Cypel M., Keshavjee S., Grasemann H. (2020). Increased Arginase Expression and Decreased Nitric Oxide in Pig Donor Lungs after Normothermic Ex Vivo Lung Perfusion. Biomolecules.

[54] Lonati C., Bassani G.A., Brambilla D., Leonardi P., Carlin A., Faversani A., Gatti S., Valenza F. (2018). Influence of ex vivo perfusion on the biomolecular profile of rat lungs. FASEB J..

[55] Hsin M.K., Zamel R., Cypel M., Wishart D., Han B., Keshavjee S., Liu M. (2018). Metabolic Profile of Ex Vivo Lung Perfusate Yields Biomarkers for Lung Transplant Outcomes. Ann. Surg..

[56] Hashimoto K., Cypel M., Juvet S.C., Saito T., Zamel R., Machuca T.N., Hsin M., Kim H., Waddell T.K., Liu M. (2018). Higher M30 and high mobility group box 1 protein levels in ex vivo lung perfusate are associated with primary graft dysfunction after human lung transplantation. J. Hear. Lung Transplant..

[57] Elgharably H., Okamoto T., Ayyat K.S., Niikawa H., Meade S., Farver C., Chan E.R., Aldred M.A., McCurry K.R. (2020). Human Lungs Airway Epithelium Upregulate MicroRNA-17 and MicroRNA-548b in Response to Cold Ischemia and Ex Vivo Reperfusion. Transplantation.

[58] Yeung J.C., Zamel R., Klement W., Bai X.-H., Machuca T.N., Waddell T.K., Liu M., Cypel M., Keshavjee S. (2018). Towards donor lung recovery-gene expression changes during ex vivo lung perfusion of human lungs. Am. J. Transplant..

[59] Wong A., Zamel R., Yeung J., Bader G.D., Dos Santos C.C., Bai X., Wang Y., Keshavjee S., Liu M. (2020). Potential therapeutic targets for lung repair during human ex vivo lung perfusion. Eur. Respir. J..

[60] Johnston M.R., Christensen C.W., Minchin R.F., Rickaby D.A., Linehan J.H., Schuller H.M., Boyd M.R., Dawson C.A. (1985). Isolated total lung perfusion as a means to deliver organ-specific chemotherapy: Long-term studies in animals. Surgery.

[61] Johnston M.R., Minchin R., Shull J.H., Thénot J.P., McManus B.M., Terrill R., Boyd M.R. (1983). Isolated lung perfusion with adriamycin. A preclinical study. Cancer.

[62] Beckers P.A.J., Versteegh M.I., Van Brakel T.J., Braun J., Van Putte B., Maat A.P., Vergauwen W., Rodrigus I., Hengst W.D., Lardon F. (2019). Multicenter Phase II Clinical Trial of Isolated Lung Perfusion in Patients With Lung Metastases. Ann. Thorac. Surg..

[63] Hengst W.A.D., Hendriks J., Balduyck B., Rodrigus I., Vermorken J.B., Lardon F., Versteegh M.I., Braun J., Gelderblom H., Schramel F.M. (2014). Phase II Multicenter Clinical Trial of Pulmonary Metastasectomy and Isolated Lung Perfusion with Melphalan in Patients with Resectable Lung Metastases. J. Thorac. Oncol..

[64] Dos Santos P.R., Iskender I., Machuca T., Hwang D., Deperrot M., Liu M., Keshavjee S., Waddell T.K., Cypel M. (2014). Modified in vivo lung perfusion allows for prolonged perfusion without acute lung injury. J. Thorac. Cardiovasc. Surg..

[65] Wang H., Bodenstein M., Markstaller K. (2008). Overview of the Pathology of Three Widely Used Animal Models of Acute Lung Injury. Eur. Surg. Res..

[66] Guenthart B.A., O’Neill J.D., Kim J., Queen D., Chicotka S., Fung K., Simpson M., Donocoff R., Salna M., Marboe C.C. (2019). Regeneration of severely damaged lungs using an interventional cross-circulation platform. Nat. Commun..

[67] Byler M.R., Haywood N., Money D.T., Zhang A., Beller J.P., Charles E.J., Chancellor W.Z., Dahl J., Ta H., Zhao Y. Two hours of protocol-driven in vivo lung perfusion improves lung function in sepsis model of acute respiratory distress syndrome. Presented at the 100th Annual Meeting (Virtual Event) of the American Association for Thoracic Surgery.

[68] Calfee C.S., Delucchi K., Parsons P.E., Thompson B.T., Ware L.B., Matthay M.A., Network N.A. (2014). Subphenotypes in acute respiratory distress syndrome: Latent class analysis of data from two randomised controlled trials. Lancet Respir. Med..

[69] Luo P., Liu Y., Qiu L., Liu X., Liu D., Li J. (2020). Tocilizumab treatment in COVID-19: A single center experience. J. Med. Virol..

[70] Ramadan K., Del Sorbo L., Cypel M. (2019). In vivo lung perfusion as a platform for organ repair in acute respiratory distress syndrome. J. Thorac. Dis..

